# Exercise training improves physical fitness in patients with pulmonary arterial hypertension: a systematic review and meta-analysis of controlled trials

**DOI:** 10.1186/s12890-015-0031-1

**Published:** 2015-04-22

**Authors:** Roselien Buys, Andrea Avila, Véronique A Cornelissen

**Affiliations:** Department of Rehabilitation Sciences, Tervuursevest 101, B 1501, B-3001, Leuven, Belgium

**Keywords:** Pulmonary arterial hypertension, Exercise training, Exercise capacity

## Abstract

**Background:**

Pulmonary arterial hypertension (PAH) is a progressive disorder characterized by hypertension in the pulmonary arteries. PAH leads to symptoms such as shortness of breath, dizziness, leg edema and chest pain, impacting heavily on quality of life. The aim of this systematic review and meta-analysis was to determine the effect of exercise training to improve physical fitness and functionality in patients with PAH.

**Methods:**

A search was conducted for controlled trials using the databases Medline, Embase, SPORT Discus and Cochrane Central Register of Controlled Trials. Studies were included if at least 80% of the participants presented with group 1 PAH and if the intervention consisted of an exercise training program of at least 3 weeks duration. Data were extracted on study quality, participant and exercise intervention characteristics, and outcomes. Data were pooled by the generic inverse variance method using random effect models and were expressed as weighted means and 95% confidence intervals (CI).

**Results:**

Of 110 identified abstracts, 5 studies with 106 patients (exercise: 53; control: 53; mean age 49.7 years) were included. Disease severity ranged from mild to severe; 96 patients suffered from PAH, 10 patients had chronic thromboembolic pulmonary hypertension. Exercise training led to an increase in 6 minute walk distance (72.5 m; 95% CI 46.0 m to 99.1 m; p < 0.0001) and peak oxygen uptake (2.16 mL/kg/min; 95% CI 2.16 to 3.93; p = 0.02). No severe adverse events during exercise were reported.

**Conclusions:**

Our findings suggest that an exercise training program positively influences exercise tolerance and functional capacity in patients with PAH.

**Electronic supplementary material:**

The online version of this article (doi:10.1186/s12890-015-0031-1) contains supplementary material, which is available to authorized users.

## Background

Pulmonary arterial hypertension (PAH) is characterized by changes in mostly the distal pulmonary arteries. This causes an increase in pulmonary vascular resistance, which in turn restricts the blood flow through the pulmonary circulation [1,2]. In order to maintain the blood flow, the pulmonary artery pressure increase which leads to right ventricular overload, hypertrophy and dilatation [1]. The physiologic response of the pulmonary vasculature to exercise is different from normal in individuals with PAH and they generally show a reduced exercise capacity [3]. Advances in the understanding and management of pulmonary arterial hypertension have enabled earlier diagnosis and improved prognosis. However, despite best available therapy, symptoms of exertional dyspnea and fatigue are commonly reported and result in a reduced capacity to perform daily activities and impaired quality of life.

Exercise training has demonstrated efficacy in individuals with other respiratory and cardiovascular diseases [4-7]. Historically, however, exercise training has not been utilized as a form of therapy in PAH due to the perceived risk of sudden cardiac death and the theoretical possibility that exercise would lead to a worsening of pulmonary vascular hemodynamics and deterioration in right heart function. Now, with the advances in pharmacological management, determining the safety and benefits of exercise training in this population has become more relevant. The Task Force for the Diagnosis and Treatment of Pulmonary Hypertension of the European Society of Cardiology and the European Respiratory Society therefore state that more data are required in order to be able to make recommendations regarding physical activity and supervised rehabilitation in patients with PAH [1]. Recently, some studies of supervised and home-based exercise training in PAH have been published and it seems worthwhile to summarize their results in order to provide some evidence for the clinicians to rely on.

Therefore, the aim of this systematic review was to apply the meta-analytic approach in controlled trials in order to determine the effects of exercise training on functional status and exercise capacity.

## Methods

### Literature search

A systematic literature search was conducted in the electronic Pubmed database from its inception to December 2013 using following terms: (medical subject heading (MeSH-term)) ‘exercise’ or (MeSH term) ‘rehabilitation’ or (field term) ‘exercise training’ AND (MeSH-term) ‘pulmonary hypertension’ or (field term) ‘pulmonary arterial hypertension’; results were filtered by ‘clinical trials’ and limitations were ‘English, French, Dutch or German language’.

From this search, we only included articles specifically addressing the effects of a supervised exercise training program in patients with pulmonary hypertension. In addition, the reference lists from published original and review articles were searched manually to identify other possible eligible studies. Embase, SPORTDiscus and The Cochrane Central Register of Controlled Trials were furthermore consulted with the same search terms.

Studies were included in the meta-analysis if they were: Controlled trials 1) including a comparative control group and an intervention group; 2) with an intervention program of at least 3 weeks duration; 3) in patients groups of which at least 80% is diagnosed with PAH as defined according to the updated clinical classification of pulmonary hypertension [1]; 4) reporting pre- and post-intervention mean and standard deviation (SD) (or standard error) of 6 minute walking distance in intervention and control groups or mean change and standard deviation (or standard error) in intervention and control groups; 5) published in a peer-reviewed journal up to December 2013. Exclusion criteria included any studies not meeting all criteria described above.

### Outcomes

The primary outcome measure was change in 6 minute walking distance (6MWD). Secondary outcomes included changes in peak oxygen uptake (peak VO_2_), peak heart rate and World Health Organization Functional Class (WHO-FC).

### Data extraction

A specific developed data extraction sheet was used to extract data on study source, study design, study quality, sample size, characteristics of the participants, exercise interventions, and the different outcomes in each study included in the meta-analysis. Data were extracted by the first two authors (RB and AA) independently from each other. The overall agreement rate prior to correcting discrepant items was 0.64. Disagreements were resolved by consensus.

### Study quality

Study quality was assessed using an adapted PEDro-scale [8]. This 11-item questionnaire is designed to collect data on eligibility criteria, random allocation, concealed allocation, similarity of baseline values, blinding of therapists and/or assessors, key outcomes, intention-to-treat analysis, between group differences, and point and variability measures. The quality criteria ‘blinding of participants’ and ‘blinding of therapists’ were omitted, since they were not applicable in the included intervention studies. All questions were binary. The minimum score was 0 and the maximum was 8, with a higher number reflecting a better study quality. The PEDro-scale has been reported to be valid and reliable [9,10]. Using Cohen’s kappa statistic, overall inter-rater agreement was 0.73. Disagreements were resolved by consensus. Trials were not excluded based on quality.

### Statistical analysis

Excel 2010 and Review Manager Software (RevMan 5.1; Cochrane Collaboration, Oxford, UK) were used for statistical analyses. Descriptive data are reported as mean ± standard deviation (SD) or median and range. The mean baseline values were calculated by combining mean values from the intervention and control groups, weighted by the number of participants in the final analysis in each study group.

Effect sizes for primary and secondary outcomes were calculated by subtracting the pre-intervention value from the post-intervention value for both study groups. The net treatment effect was then obtained by subtracting the change score difference in the control group from the change score difference of the intervention group. Review Manager Software calculated the variances from the inserted pooled SDs of change scores in the intervention and control groups. However, some studies reported only the SDs or standard errors of mean at baseline and post-intervention. Therefore, change scores SDs that were missing in these studies were calculated from pre and post SD values, using the following formula: SDchange = √[(SDpre)^2^ + (SDpost)^2^ − 2 × corr(pre,post) × SDpre × SDpost] [11], for which a correlation coefficient of 0.5 between the pre and post values was assumed. The primary and secondary outcomes were combined using random-effects models, a preferred approach that incorporates heterogeneity into the model [12,13]. Each effect size was weighted by the inverse of its variance. The results are reported as weighted means and 95% confidence intervals (CI). Two sided tests for overall effects were considered significant at p ≤ 0.05.

Statistical heterogeneity among the trials was assessed using Cochrane’s Q statistic and an alpha value for statistical significance of 0.10 indicated significant heterogeneity. In addition, we report I^2^ statistics, which assesses consistency of treatment effects across trials. I^2^ between 25% and 50% represents small amounts of inconsistency, whereas between 50-75% and >75% represents medium to large amounts of heterogeneity [14]. To examine the influence of each study on the overall results, sensitivity analyses were also performed with each study deleted from the model once. Finally, funnel plots were used to assess the potential of small publication bias. Funnel plot asymmetry was evaluated by use of Begg and Egger tests, and a significant publication bias was considered if the p value was <0.10. The trim and fill computation was used to estimate the effect of publication biases on the interpretation of the results.

## Results

### Literature search

**Figure 1:**
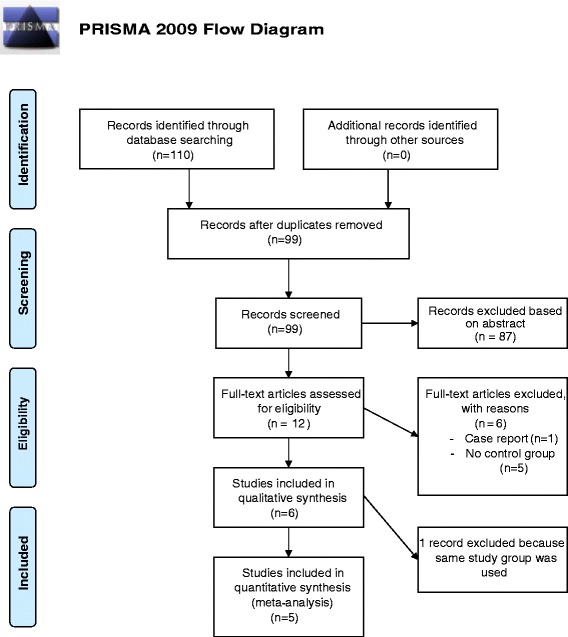
PRISMA flow chart of the literature search. *From*: Moher D, Liberati A, Tetzlaff J, Altman DG, The PRISMA Group (2009). Preferred Reporting Items for Systematic Reviews and *Meta-Analyses*: The PRISMA Statement. PLoS Med 6(6): e1000097. doi:10.1371/journal.pmed1000097 For more information, visit www.prisma-statement.org.

A PRISMA flow diagram of the literature search and selection is presented in Figure 1. From 110 potentially relevant studies retrieved from the search, only 6 trials met our inclusion criteria. However, two studies [15,16] used the same population and intervention, of which we included the most complete publication in terms of primary and secondary outcomes [15].

### Characteristics of the participants and study designs

A summary of the included studies is provided in Table 1. Four of the five studies were randomized clinical trials, the other one was a non-randomized clinical trial. Four studies used a parallel design; the remaining one used a cross-over design. All studies were published between 2006 and 2013. The exercise intervention and control groups consisted each of 53 participants. The results of the adapted PEDro-scale, used to evaluate study quality, are presented in Table 2. The median PEDro score was 6, with a range from 3 [17] to 8 [18].

**Table 1:** **Description of the patient populations and characteristics of the exercise training programs**

**Author**	**Year**	**Origin**	**Design**	**Patient population**	**NYHA**	**Age (year)**	**Number at baseline**	**Exercise intervention**	**Control**	**Assessment of physical fitness**
Mereles et al.	2006	Germany	Cross-over	PAH (80%) or chronic pulmonary thromboembolic disease (20%)	II - IV	50	Intervention: 15 (10 female/5 male); Control: 15 (10 female/5 male)	3 weeks in-hospital + 12 weeks home-based aerobic interval training along with resistance and respiratory exercises, 5 sessions/week, 30-60 min/session, 60-80% of peak HR	Usual care	6MWT, CPET
Martinez-Quintana et al.	2010	Spain	Parallel	PAH associated with congenital heart disease	II - IV	27.7	Intervention: 4 (2 female/2 male); Control: 4 (3 female, 1 male)	3 months supervised aerobic interval training and resistance exercise, 2 sessions/week, 30-60 min/session, 60-80% of peak HR	Usual care	6MWT
Fox et al.	2011	Israel	Parallel	PAH (91%) or chronic thromboembolic PH (9%)	II - III	51.5	Intervention: 11 (10 female/1 male); Control: 11 (5 female, 6 male)	3 months supervised continuous aerobic and resistance exercise, 2 sessions/week, 60 min/session, 60-80% of peak HR	Usual care	6MWT, CPET
Ley et al.	2013	Germany	Parallel	PAH (80%) or chronic inoperable tromboembolic PH (20%)	II - III	50.5	Intervention: 10 (8 female/2 male); Control: 10 (6 female, 4 male)	3 weeks supervised aerobic interval training along with resistance and respiratory exercises, 5 sessions/week, 30-60 min/session, 60-80% of peak HR	Usual care	6MWT
Chan et al.	2013	USA	Parallel	PAH	I - IV	54.4	Intervention: 13 (13 female/0 male); Control: 13 (13 female, 0 male)	10 weeks supervised treadmill walking, 3 sessions/week, 30-45 min/session, 70-80% of heart rate reserve + patient education	Patient education only	6MWT, CPET

*6MWT = 6-minute walking test; CPET = cardiopulmonary exercise testing; PAH = pulmonary arterial hypertension; PH = pulmonary hypertension.*

**Table 2:** **PEDro-scores for the intervention trials included in the meta-analysis**

**Study (year)**	**Eligibility criteria**	**Randomly allocated**	**Allocation concealed**	**Baseline similar**	**Blinding assessors**	**Key outcome 85%**	**Intention to treat**	**Between group**	**Point and variability measure**	**Total PEDro score**
Mereles (2006)	1	1	1	1	1	1	1	1	1	8
Martinez-Quintana (2010)	1	0	0	0	0	1	0	0	1	2
Fox (2011)	1	0	1	0	1	1	1	1	1	6
Ley (2013)	1	1	0	1	0	1	0	1	1	5
Chan (2013)	1	1	1	1	1	0	1	1	1	7

In total, 5 patients dropped out from the studies, resulting in a total of 101 participants (28 men) that could be included in the final analysis: 50 of them completed an exercise intervention and 51 were control subjects. Four studies included a ‘usual care’ control group (17-20) and in one study, control group participants received an educational program [15].

Baseline characteristics of the study participants are summarized in Table 3. All trials included both men and women, except for Chan who, by chance, included only women [15].

**Table 3:** **Baseline characteristics of the study participants**

**Variable**	**Exercise**	**Control**
Patients (n)	53 (40 F, 10 M)	53 (35 F, 18 M)
Mean age (years)	48.5	50.8
Pulmonary hypertension diagnosis (n)		
PAH	50	46
Ideopathic PAH	12	14
Hereditary PAH	0	1
PAH associated with congenital heart disease	5	4
PAH associated with connective tissue disease	14	14
PAH associated with portal hypertension	1	1
Drug induced PAH	0	1
PAH subtype not specified	16	11
Chronic thromboembolic pulmonary hypertension	3	7
WHO-FC (n)		
Class I	1	0
Class II	12	13
Class III	24	28
Class IV	2	1
Not reported (Fox et al.)	11	11
Mean pulmonary artery pressure (mmHg)*	49	47.1
6 minute walking test distance (m)	401	408

*n = number; PAH: pulmonary arterial hypertension; WHO-FC = World Health Organization Functional Class.*

*Not reported in Martinez-Quintana et al, mean of 4 remaining studies.

### Intervention characteristics

Table 1 also provides a description of the intervention characteristics of each trial. Study duration ranged between 3 and 15 weeks (median 12). The frequency of exercise training varied between 2 and 5 sessions weekly (median 3), with an average duration of 46 min per session (range 30-60). The average intensity was between 60 and 80% of peak heart rate in four studies [17-20], and 70-80% of heart rate reserve in the remaining study [15]. Mode of exercise was aerobic interval training along with resistance exercises in 3 trials [17,18,20], supplemented with respiratory training in two of them: Fox et al. adopted a training program with continuous aerobic exercises and resistance exercise [19] whereas Chan et al. used intensive treadmill exercise training [15].

### Assessment of primary and secondary outcomes

In all included studies, the 6-minute walk test was performed in accordance with appropriate guidelines [21]. Peak VO_2_ and peak heart rate were measured in three studies using a graded maximal exercise test on an upright cycle ergometer [19], on a treadmill [15] or on a supine cycle ergometer [18]. Four out of the five studies reported the WHO-FC before and after the intervention. Mean effect sizes and Z-scores are summarized in Table 4.

**Table 4:** **Effect sizes of primary and secondary outcomes of the meta-analysis**

**Variable**	**Study groups**	**n**	**Mean effect size (95% CI)**	**Z (p)**
6 minute walking test distance (m)	5	101	72.17 [45.71, 98.62]	5,35 (<0,0001)
Peak oxygen uptake (mL/kg/min)	3	73	2.14 [0.40, 3.88]	2,41 (0,02)
Peak heart rate (beats/min)	3	75	2.11 [-4.59, 8.81]	0,62 (0,54)
WHO-FC	4	78	-0.24 [-0.44, -0.04]	1,57 (0,12)

*n = number; WHO-FC = World Health Organization Functional Class; sPAP = peak systolic pulmonary artery pressure.*

### Changes in 6MWD

**Figure 2:**
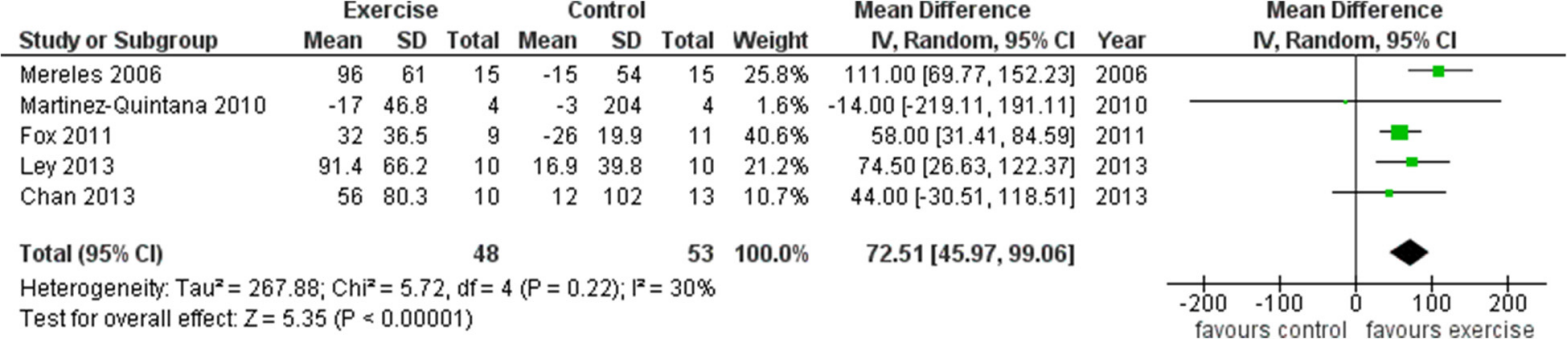
Forest plot of the average net changes and corresponding 95% confidence intervals for 6 minutes walking distance.

As shown in Figure 2, 6MWD increased significantly following exercise with a mean increase of 72.2 m (95% CI 45.7 to 98.6; p < 0.001); heterogeneity and inconsistency were small (Q = 5.72, p = 0.22; I^2^ = 30%). When each trial was deleted from the model once, the results remained statistically significant.

### Secondary outcomes

**Figure 3:**
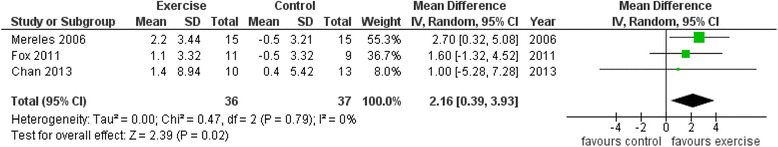
Forest plot of the average net changes and corresponding 95% confidence intervals for peak oxygen uptake.

Figure 3 shows the mean changes in peak VO_2_ with training. Peak VO_2_ increased significantly by exercise training (p = 0.02). However, when the largest study [18] was deleted from the model, statistical significance could not be met anymore.

A simultaneous change in peak heart rate due to training could not be documented (p = 0.54).

Two out of the four studies who reported WHO-FC, documented a functional improvement as assessed by this instrument, but overall no significant reduction of WHO-FC was induced by exercise training (p = 0.12).

### Publication bias

According to the tests of Begg and Egger, funnel plots of 6MWD and peak VO_2_ did not show any signs of significant publication bias (p > 0.5 for all). Duval and Tweedie’s trim and fill method indicated that one (for 6MWD) or two (peak VO_2_) studies could be missing at the right side of the mean effect size. Adjustment of the data for these missing data did not change the results. (see Additional files 1 and 2).

## Discussion

This systematic review and meta-analysis is the first to focus on the effect of exercise training on physical fitness and functional capacity in patients with PAH. The analysis, in which 5 studies could be included, showed that exercise training can result in clinically relevant improvements of physical and functional capacity.

The idea that exercise training programs may potentially be beneficial for patients with PAH became only recently accepted [1]. Exercise based rehabilitation for patients with PAH is now considered as a new adjunct treatment option in order to improve their functional capacity and quality of life. Following here, some proof of concept studies have been performed; most of them showing significant beneficial effects of exercise training on functional capacity and/or exercise capacity, but not all (Chan [15], Martinez [17], Fox [19] for peak VO_2_). Hence, the existing evidence is inconclusive and insufficient to result in the implementation of exercise programs in routine clinical care for patients with PAH. By pooling data from published controlled trials, we aimed to increase the statistical power and resolve the uncertainty. We could show that exercise training is effective for improving both functional and exercise capacity.

In todays practice the 6MWD is most often used as an endpoint to assess the benefit of therapies in PAH. It is a measure of functional capacity. This meta-analysis documented that an exercise training program of around 12 weeks can significantly improve the 6MWD with 72 m on average. This gain in walking distance is larger than that reported for patients with chronic obstructive pulmonary disease [22,23] or chronic heart failure [24]. Whereas it remains to be determined which minimum increase in 6MWD has to be reached in patients with PAH in order to obtain a clinically relevant improvement, a change of more than 50 meters has been proposed as representative of a clinically meaningful change in most disease states [25]. On the other hand, short-term improvements in 6MWD due to medical treatment could so far not be related to an improved longer-term prognosis [26] even though a baseline distance less than approximately 330 m on the 6MWD has been shown to be associated with increased mortality in PAH [27]. In line with these results, Golpe and colleagues in non-group 1 PAH patients reported that a distance less than 400 m was predictive of a significantly worse prognosis [28]. In our study, mean baseline 6MWD was already more than 400 meter, however it is anticipated that the average observed gain in 6MWD of 72 m due to exercise training, will have shifted a part of the weaker patients towards a 6MWD larger than 400 m and thus towards a more favorable prognosis.

Overall, cardiopulmonary exercise testing is the preferred method for assessing changes associated with exercise training [29]. Despite being less often used, cardiopulmonary exercise testing should also be considered as the gold standard measurement of exercise capacity in patients with PAH [30]. Only three of the included studies reported exercise training related changes in oxygen uptake. Nevertheless, this was already sufficient to document a small but significant increase in peak VO_2_ following exercise training. This result is also in line with several other small-scale non-controlled studies [31] and adds further evidence to support the use of exercise training to increase peak VO_2_ in this patient population. Whereas the clinical significance of these small improvements (+2.6 ml/min/kg peak VO_2_) in this population remains inconclusive, Groeppenhof et al. showed that among patients with PAH, survivors have larger increases in exercise capacity compared to non-survivors [32].

The underlying mechanisms causing these training effects might be of both central pulmonary and cardiovascular as well as peripheral origin, but need to be further clarified. In addition, whether baseline and especially training-related changes in objective exercise capacity reflect a better prognosis both in short and longer term of patients with PAH still needs to be determined [30].

Exercise capacity is closely related to the ability to perform normal daily life activities. In turn, this closely relates to quality of life. Exercise tests can indirectly quantify shortness of breath and fatigue, two of the most common symptoms of PAH. Therefore, the change in 6MWD and peak VO_2_ from baseline to the end of an exercise training program might suggest symptomatic improvement over that time-period [33]. Although we could only show a tendency (p = 0.12) toward a functional improvement by means of the WHO-FC scale, this was only based on four studies and more studies are warranted. Moreover, the effect of exercise training on quality of life in these patients needs further research due to the importance for both clinicians and patients.

### Limitations

There are several limitations to our work. Although meta-analyses are no substitute for large well-designed controlled trials, the meta-analytical technique is probably the best method to systematically review previous work. Advantages are the greater precision of the estimates and the enhanced statistical power. In order to provide the most accurate summary of the effects of exercise training for PAH, the PRISMA statement was carefully followed [34]. A potential disadvantage of a meta-analysis is the heterogeneity of studies for instance due to variations in the exercise programs and the differences between patient populations [35]. Although random-effects models which incorporate heterogeneity into their model were used, some caution might be warranted with the interpretation given also the small number of studies and the small sample sizes. Further, as there were only 3 randomized trials, we also included 2 non-randomized studies. The results of those may be affected by confounding bias due to the absence of random assignment.

## Conclusions

The findings from this systematic review and meta-analysis suggest that an exercise training program positively influences exercise tolerance and functional capacity in patients with PAH. Physicians need to become aware of the positive effects of this non-pharmacological intervention that can improve the integrated care of patients with PAH. Given the small number of available studies and the small sample sizes, we believe that this topic deserves a more in-depth investigation. Well-designed randomized clinical trials investigating the effects of exercise training on exercise capacity and patient outcomes are needed to confirm our results.

## Additional files

Additional file 1:
**Funnel plots for 6 minute walking distance.**
Additional file 2:
**Funnel plots for peak oxygen uptake.**

## Abbreviations

PAH: Pulmonary arterial hypertension
CI: Confidence intervals
MeSH: Medical subject heading
6MWD: 6 minute walking distance
peak VO_2_: Peak oxygen uptake
WHO-FC: World Health Organization Functional Class
SD: Standard deviation
