# Supplementary figures and images for: Exercise training improves physical fitness in patients with pulmonary arterial hypertension: a systematic review and meta-analysis of controlled trials
Source: BMC Pulm Med. 2015 Apr 22;15:40. doi: 10.1186/s12890-015-0031-1 (PMC4423624; doi:10.1186/s12890-015-0031-1)

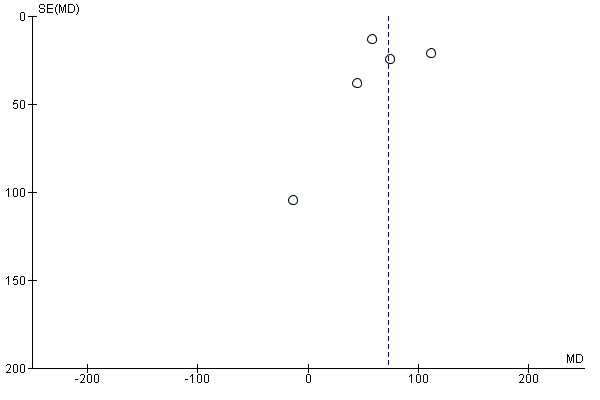

Supplement: Additional file 1: — Funnel plots for 6 minute walking distance. [file 12890_2015_31_MOESM1_ESM.png]

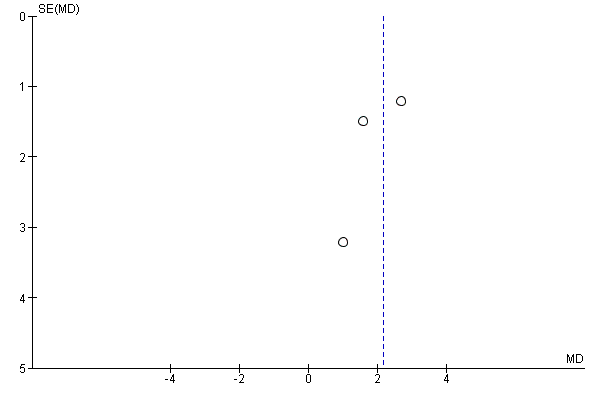

Supplement: Additional file 2: — Funnel plots for peak oxygen uptake. [file 12890_2015_31_MOESM2_ESM.png]
